# Can the unnecessary operations for suspected thyroid nodules be avoided by the combined use of the strain ratio and elastography score?

**DOI:** 10.1016/j.bjorl.2020.05.017

**Published:** 2020-06-25

**Authors:** Orhan Görgülü, Feride Fatma Görgülü, Ayşe Selcan Koç

**Affiliations:** a University of Health Sciences, Adana Health Practice and Research Center, Department of Otorhinolaryngology, Adana, Turkey; b University of Health Sciences, Adana Health Practice and Research Center, Radiology Department, Adana, Turkey

**Keywords:** Thyroid nodule, Strain elastography, Strain ratio, Elastography score, Malignancy

## Abstract

**Introduction:**

Only 5%–15% of thyroid surgical specimens are reported as malignant. Most of the operations are performed due to suspicion of malignancy as a result of fine needle aspiration biopsy but invasiveness, non-diagnostic results and potential repeat biopsies are disadvantages of fine needle aspiration biopsy.

**Objective:**

The aim of this study was to investigate the effectiveness of simultaneously using both the strain ratio and elasticity score in the differential diagnosis of thyroid nodules, as well as to assess the compatibility of these two methods.

**Methods:**

A total of 144 nodules were included in the study. The final histopathologic diagnosis was used as the reference standard. The area under the curve sensitivity, specificity, and cut-off values of the strain ratio and elasticity score were determined using receiver operating characteristic curve analysis. The compatibility and comparison of strain ratio and elasticity score were also performed.

**Results:**

Twenty eight nodules (19.4%) were malignant. The strain ratio and elasticity score results were found to be significantly successful in predicting thyroid malignancy (*p* < 0.001 for both). Moreover, the area under the curve for the strain ratio and elasticity score were found to be 0.944 and 0.960, respectively. The diagnostic accuracy of the elasticity score was found to be superior to that of the strain ratio, but this difference was not statistically significant (*p* = 0.456). When the compatibility of the strain ratio and elasticity score was examined, the two evaluations were revealed to be statistically consistent with each other (Kappa = 0.767; *p* < 0.001). When the strain ratio and the elasticity score were used together, the specificity of capturing the correct diagnosis increased from 84.5% to 93.1%.

**Conclusion:**

When the strain ratio an elasticity score were used together for the differential diagnosis of thyroid nodules, more accurate results were obtained. Thus, combining both methods may be a promising alternative to fine needle aspiration biopsy in order to prevent unnecessary surgical interventions for suspected thyroid nodules.

## Introduction

Thyroid nodular disease is commonly detected in geographic areas with known iodine deficiency. The thyroid nodules can be detected via palpation in 4%–7% of cases using Ultrasonography (US) in 27%–67% of the general adult population.^1^, ^2^ Additional imaging modalities are needed to detect malignancies that comprise approximately 5%–15% of all thyroid nodules, since the number of nodules increases with the development of such medical imaging.^3^

Ultrasonography (US) provides valuable information regarding the presence of malignancy, but it is not always successful in distinguishing between benign and malignant lesions. Therefore, Fine Needle Aspiration Biopsy (FNAB) is required for those with conspicuous ultrasonographic features^4^, ^5^; however, FNAB is an invasive procedure and has a 90% diagnosis sensitivity for thyroid cancer.^6^, ^7^

Palpation is the most traditional and basic detection method for the diagnosis of thyroid lesions. Nodules with suspected malignancy usually have a harder structure than others; however, palpation is a subjective detection method, since it can be impacted by the location and size of the nodule as well as the experience of the clinician.^8^ Strain Elastography (SE) aims to provide a real-time, noninvasive characterization of tissue stiffness in the same session as conventional ultrasound imaging and without the use of a contrast agent.^9^ This method is based on the fact that hard tissues are associated with an increased risk of malignancy. On average, benign thyroid nodules are 1.7 times and malignant thyroid nodules are 5 times stiffer than normal thyroid tissue. Moreover, the elastic coefficients and color scale obtained from this study enable a different analysis of the examined lesion and surrounding tissues, making it possible to quantify nodule stiffness in comparison to the surrounding parenchyma. This method has been proposed to differentiate benign thyroid nodules from malignant ones, and several authors have investigated its usefulness.^10^ SE is also used to evaluate different clinical situations, such as the distinction between autoimmune thyroid lesions, breast tumors, and so on.^11^, ^12^ There are two methods for evaluating the diagnostic effectiveness of SE: the elasticity score, which employs qualitative assessment via color coding, and the strain ratio, which is a semi-quantitative evaluation using numerical parameters.^13^

Previous studies have compared the clinical value of both the elasticity score and/or strain ratio in the differential diagnosis of thyroid nodules. The aim of this study was to once again evaluate the effectiveness of the two techniques, to assess their compatibility, and to investigate the effectiveness of using these methods together rather than separately.

## Methods

This prospective study was conducted in compliance with the Helsinki Declaration and the good clinical practice guidelines from the Ministry of Health of Turkey. The study was approved by the local ethics committee of the Adana Numune Research and Training Hospital in Adana, Turkey (EK 2013/22). All patients provided written, informed consent to participate in the study. Informed consent from patients under 18 years of age was provided by their parents. Histopathologic results were used as the reference standards.

### Study population

Patients who were admitted to the otorhinolaryngology and general surgery clinics in Adana Numune Research and Training Hospital (Adana, Turkey) for thyroidectomy according to preoperative clinical evaluation were included in the present study, which resulted in a total of 144 nodules from 123 patients.

The existence of pure cystic lesions, insufficient normal tissue surrounding the measured nodule, isthmic nodules, nodules larger than 40 mm, rough calcification and autoimmune thyroid disease were all exclusion criteria. The inclusion criterion was the presence of single or multiple nodules ≤40 mm and no history of the thyroid operation. Demographic and laboratory data, including age, gender and FNA results were also recorded. The final histopathologic diagnoses were used as the reference standards.

### Imaging methods

Both B-mode ultrasound and strain elastography were performed using an Aplio 500 ultrasound machine (Toshiba Medical Systems, Co., Ltd., Otawara, Japan) with linear 4.8–11 MHz transducers as well as elastography software. Two radiologists, both with thyroidal imaging experience of more than eight years and thyroid elastography experience of at least two years performed all the measurements. Specifically, one radiologist performed the strain elastography, measuring SR and image recording (FFG), while the other determined the elastography score over recorded image without being aware of the SR value and B-mode US view (ASK). The radiologists were blinded to the clinical findings, laboratory results, FNAB results and the suspected differential diagnosis of the patients.

**Figure 1:**
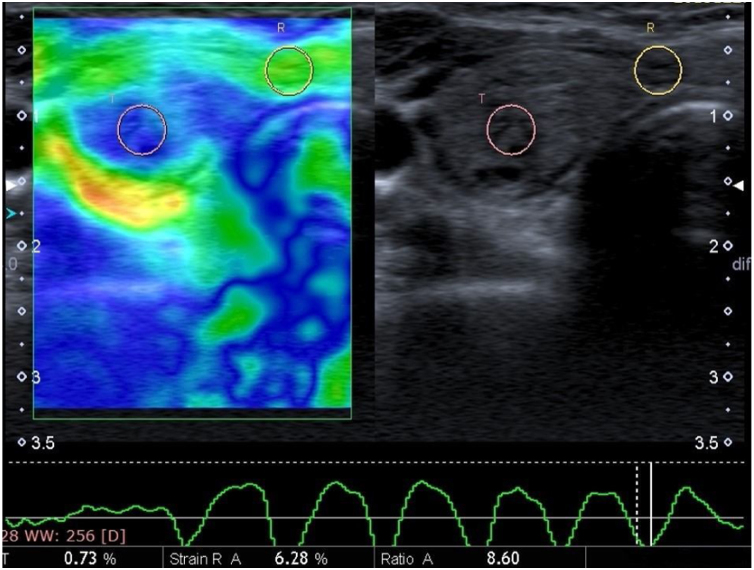
Dual screen B-mode ultrasound imaging (right) and US elastogram (left) reveal large proportions of hard blue regions with a few light green areas mixed in (score of 3). The average strain ratio was 8.60. The histopathological examination revealed a papillary thyroid carcinoma.

**Figure 2:**
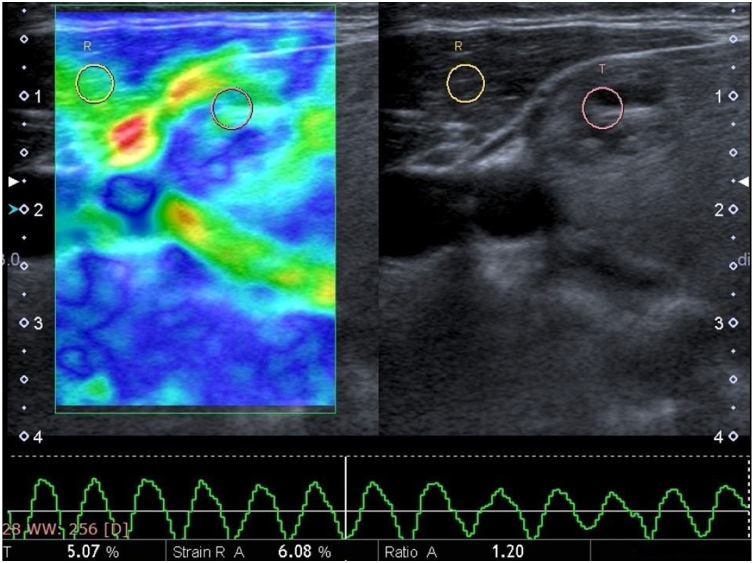
Image of a 38 year-old woman from a dual screen US elastogram. The nodule exhibits a small degree of stiffness with a mixed color of mostly green, a little blue areas consistent with a score of 2. The strain ratio was 1.20. These results indicated a benign form of the disease, and histopathology confirmed a benign nodule.

A routine B-mode ultrasound evaluation was obtained by having patients positioned on their backs with their necks slightly extended. B-mode ultrasonographic nodule features, such as size and side location (e.g., right, left), were recorded. SE was performed with a conventional ultrasound probe during a single US examination session using software connected to the machine. The probe was positioned perpendicular to the skin when pressure was applied, and lateral movement was avoided. The great cervical vessels were also avoided as much as possible. Additionally, strong initial compression was avoided due to the possibility of increasing the probability of false negative results. Patients were asked to hold their breath and not swallow during the examination in order to minimize the movement of the thyroid gland. After approximately seven to eight compression–relaxation cycles, the elastographic examinations were finalized. SR value measurements were obtained from appropriate relaxation waves on the velocity profile, and the SR was automatically calculated by the software. Each nodule was assessed based on different static images at least three times, with the mean value recorded as the final result. The radiologist performed the examination and evaluation for approximately 5–7 min for each patient. For the SR, the radiologist selected the rounded Region-Of-Interest (ROI), including the thyroid nodule and surrounding normal thyroid tissue or the ipsolateral Sternocleidomastoid Muscle (SCM), as a reference in order to provide a semi-quantitative analysis. The elastography score was also measured via the same method and determined by a color scale, ranging from green (i.e., softest components) to blue (i.e., hardest components). According to this assessment system, softer lesions are assigned lower elastography scores, while stiffer lesions are assigned higher elastography scores. The gray-scale images on the right and images of the strain elastogram on the left were displayed on the same screen for both methods (Figs. 1‒2).

**Figure 3:**
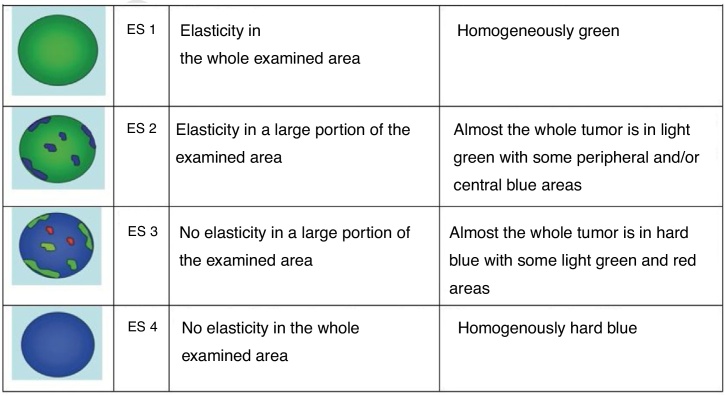
Elasticity Score (ES) of thyroid nodules and its corresponding elastographic pattern proposed be Asteria et al.^14^

Elastography score was based on a four-point scale according to the classification proposed by Asteria et al. (Fig. 3).^14^

### Statistical analysis

A Shapiro Wilk test was used to check the normality of the continuous variables, while a student’s *t*-test was used to compare the mean of the two independent groups. Descriptive statistics were expressed as mean, standard deviation, and minimum and maximum values. In the analysis of the categorical variables, a Chi-Square test was used, while a Fisher Exact test was used for comparisons of more than five observations over 20%. According to the histopathological results, the Receiver Operating Characteristic (ROC) analysis was used to determine the cut-off point for the Strain Ratio and Elastography Score and to evaluate the diagnostic test performances. The area under each curve was compared with the method proposed by DeLong et al. (1988). Descriptive statistics were expressed as the area under the curve (AUC) (95% Confidence Interval), sensitivity (95% Confidence Interval), and specificity (95% confidence interval). The Kappa statistic was calculated while investigating their conformity to the cut points calculated for the SR and ES. Since the FNAB results are categorical data, sensitivity (95% Confidence Interval), specificity (95% Confidence Interval), positive predicted value (PPV 95% Confidence Interval) and negative predicted value (NPV 95% Confidence Interval) were calculated. The statistical significance level was taken as 0.05 for all analyzes. Data analysis was performed using the free trial version of Medcalc 18.10.2.

## Results

### Clinical and demographic findings

A total of 144 nodules from 123 patients (87 females, 36 males; mean age: 45.33 ± 12.47 years; range: 15–79 years) were included in the study. The mean size of the nodules was 17.23 ± 8.01 mm (within a range of 8–40 mm). There were no statistically significant differences between benign and malign histopathology groups based on age, gender, nodule size, or nodule location (Table 1).

**Table 1:** Demographic and nodule characteristics of the study population.

	Benign	Malign	Total	*p*
Age (Mean ± SD) (Min‒Max)	45.95 ± 12.27 (15–79)	43.01 ± 13.58 (21–63)	45.33 ± 12.47 (15–79)	0.414
Gender				0.567
Female	67 (69.1%)	20 (76.9%)	87 (70.7%)	
Male	30 (30.9%)	6 (23.1%)	36 (29.3%)	
Nodule location				0.753
Right	68 (58.6%)	15 (53.6%)	83 (%57.6)	
Left	48 (41.4%)	13 (46.4%)	61 (%42.4)	
Nodule size (Mean ± SD) (Min‒Max)	17.03 ± 7.45 (8–40)	17.93 ± 4.79 (11–27)	17.23 ± 8.01 (8–40)	0.677

### Comparison of the diagnostic performance of the SR and ES

The strain ratio, elastography score and FNAB results were found to be significantly successful in differentiating benign-malign histopathology (*p* < 0.001 for three) (Table 2).

**Table 2:** Comparison of histopathological diagnosis according to strain ratio, elastography score, and FNAB results.

	Benign, n (%)	Malign, n (%)	*p*
Strain ratio ≤ 3.59	98 (84.5%)	0 (0.0%)	<0.001
Strain ratio > 3.59	18 (15.5%)	28 (100.0%)	
Total, n (%)	116 (100%)	28 (100.0%)	
Elastography score 1‒2	100 (86.2%)	0 (0.0%)	<0.001
Elastography score 3‒4	16 (13.8%)	28 (100.0%)	
Total	116 (100%)	28 (100.0%)	
FNAB benign	106 (91.4%)	1 (3.6%)	<0.001
FNAB non-diagnostic	4 (3.4%)	19 (67.8%)	
FNAB malign	6 (5.2%)	8 (28.6%)	
Total	116 (100%)	28 (100.0%)	

**Figure 4:**
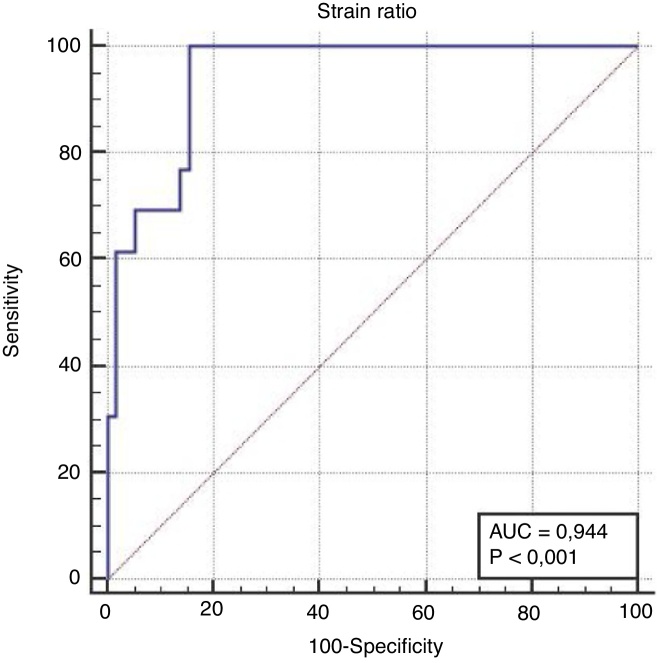
ROC curve analysis for the differential diagnosis of benign and malignant nodules using the SR. AUC was 0.944 with 100.0% sensitivity and 84.5% specificity.

**Figure 5:**
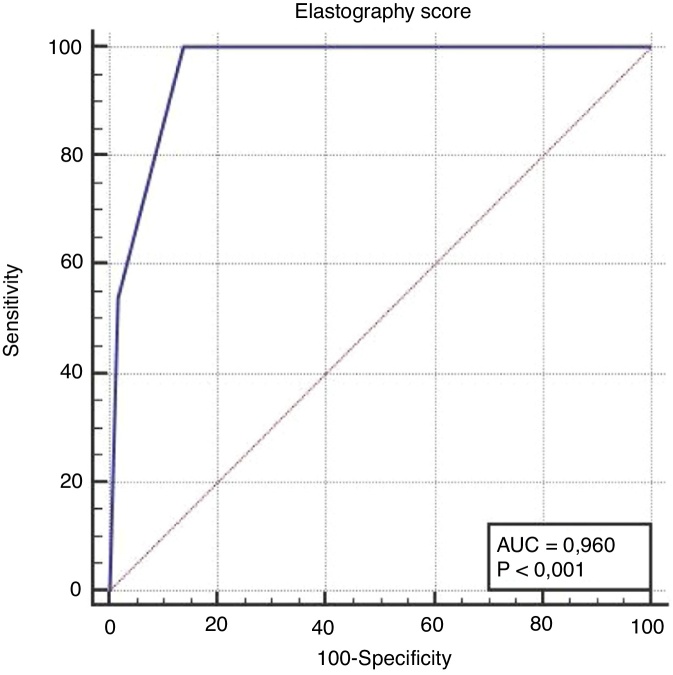
ROC curve for the ES to diagnose thyroid malignancy with an AUC of 0.960. The sensitivity and specificity were 100.0% and 86.2%, respectively.

**Figure 6:**
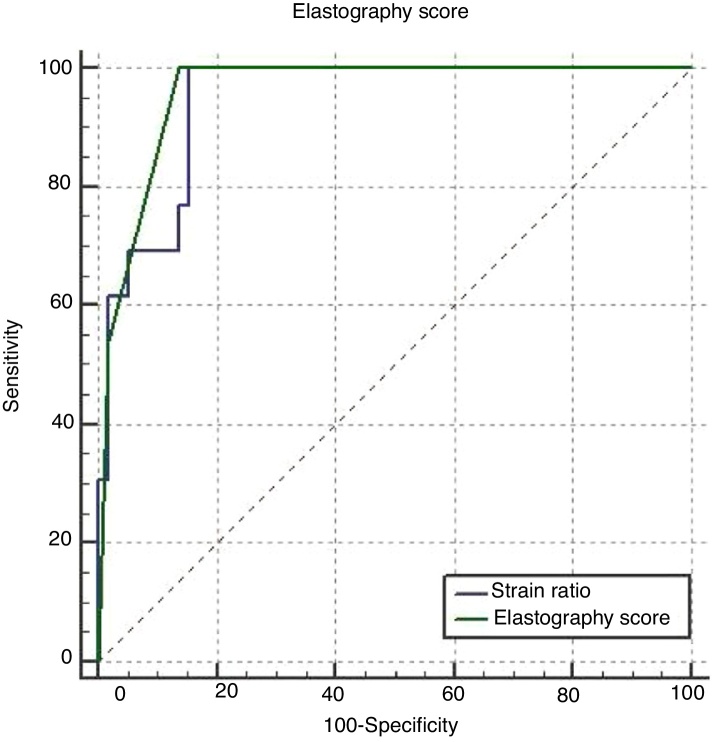
ROC curves for distinguishing between malignant and benign thyroid nodules. The blue line represents the SR ROC curve, while the green curved line represents the ES ROC curve. The difference between the AUCs was found to be 0.016 (*p* = 0.456).

In terms of distinguishing malignancy, the AUC for the strain ratio was found to be 0.944 and the cut-off point was determined to be >3.59 (*p* < 0.001). For the ES, the AUC was found to be 0.960 and the cut-off point was determined to be ≥3 (*p* < 0.001) (Table 3, Figure 4, Figure 5). When comparing the areas under the curve for the two methods, the difference was found to be 0.016, which was not statistically significant (*p* = 0.456) (Fig. 6). The diagnostic accuracy of the SR was superior to that of ES, but this difference was also not statistically significant (Table 3).

**Table 3:** Comparison of the diagnostic performance of the SR, the ES and FNAB.

	Area under curve (95% CI)	Sensitivity (95% CI)	Specificity (95% CI)	Cut off value	*p*
Strain ratio	94.4 (86.3–98.5)	100.0 (75.3–100.0)	84.5 (72.6–92.7)	>3.59	<0.001
Elastography score	96.0 (88.4–99.2)	100.0 (77.5–100.0)	86.2 (74.6–93.9)	≥3	<0.001
FNAB results	93.0 (84.6–97.0)	100 (79.1–100.0)	91.4 (81.0–97.1)	‒	<0.001
Difference between areas (SR-ES)	1.60 (−2.49 to 5,54)				0.456

### The compatibility and effect of the co-use of the SR and ES

When the compatibility of the SR and ES were examined, it was observed that the two evaluations were statistically consistent with each other (Kappa = 0.767; *p* < 0.001) (Table 4). When the SR and ES were used together in predicting thyroid malignancy, the specificity of capturing the correct diagnosis increased to 93.1% (Table 5).

**Table 4:** Compatibility of the SR and ES.

	Strain ratio ≤ 3.59	Strain ratio > 3.59	Total	Kappa value	*p*
Elastography score 1–2	92	8	100	0.767	<0.001
Elastography score 3‒4	6	38	44		
Total	98	46	144

**Table 5:** Analysis of the SR and ES together in predicting thyroid malignancy.

Classification results^a^					
		Pathology	Predicted group membership		Total
			Benign	Malignant	
Original	Count	Benign (%)	106 (91.4)	10 (8.6)	116 (100.0)
		Malignant (%)	0 (0.0)	28 (100.0)	28 (100.0)

a 93.1% of original grouped cases correctly classified according to final pathology results using the two techniques together.

## Discussion

This study demonstrated that the SR, the ES and FNAB results were significantly successful in differentiating benign-malign histopathology (*p* < 0.001 for three). There was also a positive correlation as well as compatibility between the SR values and ES scores (Kappa = 0.767; *p* < 0.001). Furthermore, the present study found that the diagnostic accuracy of the SE was superior to that of the SR; however, this difference was not statistically significant.

US examination is an appropriate method for examining thyroid nodules, but its usage prevalenc and success in distinguishing between benign and malignant thyroid nodules is relatively small. Conventional US also does not provide the necessary information regarding the stiffness of the thyroid nodule.^15^ Therefore, elastography, a non-invasive method seen as a type of electronic palpation, can be used for this purpose, since it can provide objective information about the stiffness of the tissue. Depending on the content of the nodule, thyroid lesions may exhibit different stiffness levels. Since the thyroid gland is a superficial organ, it is suitable for elastography that is also used for cervical lymph nodes, because external deformation is relatively easy to detect.^16^ However, elastography can be influenced by radiologist experience as well as degree of compression applied. Some have suggested, in previous ex vivo and in vivo studies, that there is a clear difference between the stiffness of normal thyroid tissue and thyroid tumors.^17^, ^18^

SE is a non-invasive, ultrasound-based method that provides a qualitative and semi-quantitative evaluation of tissue stiffness using conventional ultrasound probes. The measurement of tissue elasticity is based on the stress and strain of the tissue being examined. The ES and SR are used as diagnostic parameters for SE.^19^ In the present study, both methods (ES and SR) were used to evaluate the elasticity of thyroid nodules. The ES evaluation is based on four scales that assess tissue stiffness within a lesion. In this evaluation system, softer lesions are represented by lower elastography scores (green color) and stiffer lesions by higher elastography scores (blue color).^14^ Regions of interest were selected on the target lesion and the adjacent reference thyroid tissue or same side SCM for the SR. An SR of >1 reveals that the target lesion has a higher stiffness than the reference tissue. The malignancy rate of a lesion was considered to be higher with increased SR and lower with reduced SR. Benign soft-tissue lesions were assumed to be stiffer than normal tissue and softer than cancers.^20^, ^21^ Some valuable advantages of SE are its non-invasiveness, its performance simplicity, and its suitability for routine ultrasound examinations. SE methods require experienced sonographers, because they are difficult to standardize for freehand compression, and non-uniform compression can be introduced by different operators, causing potential inter -or intra-observer variation.^22^

Both the SR and ES values of malignant thyroid nodules are higher than their benign counterparts.^9^, ^23^ In some studies, SR is more successful than the grading method at identifying malignancy; it is able to provide semi-quantitative information about thyroid nodules that improves overall diagnostic confidence.^19^, ^24^ In their study, Ma et al. found that the SR exhibited a better diagnostic performance than the ES.^25^ This finding was also confirmed in a good meta-analysis, in which the SR was accepted as a better predictor of thyroid malignancy.^26^ For the identification of papillary thyroid cancer, SR was also found useful.^27^ Moreover, two subsequent studies revealed that the SR measurements had sensitivities of 82% and 97.8% and specificities of 96% and 85.7%, respectively.^9^, ^28^ In the present study, the sensitivities and specificities of the SR values were 100% and 84.5%, respectively, when the best cutoff point of 3.59 was used. The usefulness of thyroid elastography to predict thyroid cancer was demonstrated in two other studies.^18^, ^29^ In addition to these findings, the ES also exhibited promising results with a high degree of accuracy in differentiating between benign and malignant thyroid nodules in various other studies.^14^, ^30^, ^31^, ^32^ Elasticity scores 3–4 and a strain ratio >3.59 were found more frequently for malignant than benign nodules in the present study. When comparing the AUC for the two methods, ES was found to be greater than the SR evaluation (0.960 vs. 0.944, respectively), but this difference was found to be 0.016, which was not statistically significant (*p* = 0.456).

Sun et al.,^19^ in their meta-analysis, found a higher sensitivity for ES but a higher specificity for SR, with lower specificity for ES in nodules with nondiagnostic cytologic findings. However, the study by Lippolis et al.^33^ revealed the opposite results for thyroid nodules with indeterminate cytology. It should also be noted that SE is not recommended for medullary thyroid carcinoma and follicular thyroid carcinoma diagnosis due to an excess of false-negative results.^34^, ^35^

The present results confirmed the usefulness of ES and SR in detecting malignant thyroid nodules with high sensitivity and specificity. The SR used together with ES in predicting thyroid malignancy exhibited a better diagnostic performance (capturing the correct diagnosis increased to 93.1%) than the use of SR alone. This result showed that combining these methods is a significantly stronger predictor of malignancy for solid thyroid nodules. To the best of our knowledge, the present study is one of the first combining the SR and ES values as a reliable tool for the differential diagnosis of thyroid nodules.

Shrestha et al. reported that the sub-centimeter nodules had the highest malignancy rate.^36^ The American Association of Clinical Endocrinologists also recommended biopsy for nodules ≤1 cm in diameter and with suspicious US features.^37^ Nodules of 5 mm or less produce high false positive US findings and often provide insufficient cytology in FNAB.^38^ US-guided FNAB has been commonly used as the standard diagnostic tool for thyroid nodules, exhibiting good performance. Disadvantages of US-FNAB include its invasiveness, sampling errors, and inadequate non-diagnostic results for about 20%–30% of FNABs, which leads to repeat biopsies.^3^, ^7^, ^21^, ^39^ A significant percentage of thyroid nodule FNABs reveal benign findings; therefore, it is not an appropriate evaluation of all thyroid nodules due to the frequent occurrence of such nodules. It has been reported that elastography as a noninvasive method may circumvent the need for biopsy in many patients with thyroid nodules, potentially reducing the percentage of unnecessary FNABs to 53%–60.8%.^37^, ^40^ In our study, we found that the benign—malignant discrimination specificity of FNAB alone was 91.4%, whereas when SR and ES were used together, this discrimination rate was 93.1%.

The number of patients diagnosed with benign thyroid nodules as their final diagnosis is much higher than the number of patients diagnosed with malignant nodules. Due to the high number of the thyroid nodules, invasiveness, and cost increases, FNAB cannot be performed for each patient. This also means a waste of resources and money. The strain elastography (The SR and ES) has excellent diagnostic efficiency in terms of differentiating between benign and malignant thyroid nodules and can also be used to determine which nodules require FNAB and which patients require repeat FNAB.

The present study had some limitations: first, the sample size was not large enough. Secondly, patients selected for surgery were included in the study; therefore, the prevalence of malignancy in the study could be higher than that of the general population. Moreover, the compression maneuvers performed with the transducer can cause excessive stiffness, especially in superficial tissues, which could lead to misdiagnosis. To prevent this event, the present clinician, who had at least two years of experience with elastography, maintained light contact on the probe before beginning the cycles of palpations. Additionally, measurements were repeated at least three times, and mean values were used to reduce intra-observer variability. Nodules larger than 40 mm, pure cystic and shell-calcified nodules were also avoided due to difficulties in measuring their elasticities.

## Conclusions

In our study, we found that the strain elastography in which SR and ES were evaluated together, was able to distinguish benign-malignant thyroid nodules at least as accurately as or even slightly more accurately, than FNAB. We propose that this co-use can be a promising fast-resulting non-invasive alternative to FNAB whose result requires considerable time and provide the avoidance of unnecessary surgical interventions for suspected thyroid nodules. We propose that it is time for the strain elastography to take its deserved place in the diagnostic algorithm of thyroid nodules. Specifically, large, multicenter, prospective studies of a larger population are needed to measure the effectiveness of the co-use of the SR and ES in order to validate these results.

## Conflicts of interest

The authors declare no conflicts of interest.
